# CircRNAs in BALF exosomes and plasma as diagnostic biomarkers in patients with acute respiratory distress syndrome caused by severe pneumonia

**DOI:** 10.3389/fcimb.2023.1194495

**Published:** 2023-08-22

**Authors:** He Sun, Wei Gao, Rongzhang Chen, Si Chen, Xia Gu, Feilong Wang, Qiang Li

**Affiliations:** Department of Respiratory and Critical Care Medicine, Shanghai East Hospital, Tongji University, Shanghai, China

**Keywords:** severe pneumonia, acute lung injury/acute respiratory distress syndrome, exosome, circRNA, diagnostic markers

## Abstract

**Background:**

The transcriptomic studies targeting circular RNAs (circRNAs) in bronchoalveolar lavage fluid (BALF) exosomes of acute respiratory distress syndrome (ARDS) patients caused by severe pneumonia have rarely been reported. This study aimed to screen and validate abnormally expressed circRNAs in exosomes from BALF of patients with ARDS caused by severe pneumonia and then evaluate the diagnostic values of these circRNAs for ARDS.

**Method:**

BALF was collected from four patients with ARDS caused by severe pneumonia and four healthy subjects. CircRNA expression profile was obtained by microarray analysis in BALF exosomes of the discovery cohort. The differentially expressed circRNAs in BALF exosomes were verified by real-time quantitative PCR (RT-qPCR) and underwent competitive endogenous RNA (ceRNA) network construction and functional enrichment analysis.

**Results:**

A total of 629 circRNAs were differentially expressed in BALF exosomes between ARDS patients and healthy subjects. Nine differentially expressed circRNAs were validated by RT-qPCR, and seven were consistent with the results of microarray analysis. CeRNA network analysis was performed for hsa_circRNA_002809, hsa_circRNA_042882, and hsa_circRNA_104034. Functional enrichment analysis showed that the target genes were mainly associated with hypoxia-induced damage, inflammatory response, and the HIF-1 signaling pathway. Hsa_circRNA_042882 and hsa_circRNA_104034 can be regarded as promising diagnostic biomarkers for patients with ARDS caused by severe pneumonia, with remarkable sensitivity and specificity of the area under the curve of 0.8050 and 1 or 0.835 and 0.799, respectively.

**Conclusion:**

This study obtained circRNA expression profiles of ARDS patients, and hsa_circRNA_042882 and hsa_circRNA_104034 were regarded as promising diagnostic biomarkers for patients with ARDS caused by severe pneumonia.

## Introduction

Acute lung injury (ALI) and its more severe stage acute respiratory distress syndrome (ARDS) are characterized by acute inflammatory lung injuries caused by multiple etiologies, posing a serious threat to human health worldwide (Sweeney and McAuley, 2016). Recent epidemiological survey reports that the incidence of ARDS in the intensive care unit (ICU) is more than 10%, and the mortality rate is as high as 34.9%–46.1% (Bellani et al., 2016). Severe pulmonary infection is the main cause of ARDS. Current treatments for ARDS still focus on controlling the underlying disease that induces ARDS and supportive care based on improving gas exchange and preventing complications. However, due to the lack of effective drugs to improve prognosis (Thompson et al., 2017; Fan et al., 2018; Matthay et al., 2019), the treatment of ALI/ARDS presents a great challenge.

During the occurrence and development of ARDS, various immune cells, lung structural or functional cells, and their secreted or chemotactic cytokines/inflammatory mediators jointly create the inflammatory microenvironment of the lung (Steinberg et al., 1994; Westphalen et al., 2014; Morrell et al., 2019). Notably, excessive and uncontrolled inflammatory response contributes to lung tissue injury and dysfunction, which is the critical enabler in the pathophysiology of ALI/ARDS. Thus, it is essential to search for effective methods to moderately modulate the inflammatory response, thereby avoiding lung tissue damage, which may improve the prognosis of ARDS. Unfortunately, scientists have not yet found an appropriate method to regulate the excessive inflammatory response in the acute phase of ARDS.

Currently, accumulated studies have found that non-coding RNAs, including lncRNAs, microRNAs, and circular RNAs (circRNAs), play a regulating role in the development, progression, and prognosis of ARDS (Juan et al., 2018; Fanucchi et al., 2019; Xiao et al., 2020; He et al., 2021). CircRNA is a closed long-chain non-coding RNA with reverse splicing sites. Its unique closed circular structure, which makes it difficult to be cut by RNA exonuclease, is highly stable and allows for continuous and stable regulation of target genes without off-target effects. CircRNAs have been proven to regulate the transduction of signaling pathways through multiple mechanisms such as competitive endogenous RNA (ceRNA) mechanisms, acting as miRNA sponges and binding proteins (Zheng et al., 2016; Xiao et al., 2020). CircRNA can also interact with proteins to regulate the transduction of signaling pathways. Recent studies on viral infections have found that specific circRNAs can mediate the host antiviral response by activating retinoic acid-induced gene protein I (RIG-I), an important molecule in the body’s innate immune defense. In addition, circRNA is able to enter the Ribosome to translate peptides due to its Internal Ribosome Entry Site (IRES) and its open reading frame (ORF) (Zheng et al., 2016; Wang et al., 2017; Bao et al., 2019; Xiao et al., 2020). Different scholars have observed differentially expressed circRNAs in lung tissues of ALI model animals and plasma of patients with traumatic lung injury using circRNA microarray or sequencing technology (Wan et al., 2017; Bao et al., 2019; Jiang et al., 2020). Some researchers silenced circ_0054633 to alleviate lipopolysaccharide (LPS)-induced ALI by inhibiting NF-κB activation (Yang et al., 2021). These results provide an experimental basis for the molecular mechanism of circRNA regulating ARDS. However, the loading and transportation of circRNA is also an important guarantee to realize its function.

A recent study has also demonstrated that exosomes from different cells can regulate the function of target genes and proteins by carrying specific lncRNAs, miRNAs, and DNAs (Jiang et al., 2020). Furthermore, *in vitro* and *in vivo* studies have revealed that exosomes from different cells can influence pulmonary function by the relevant inflammatory mediators or microRNAs, thereby affecting the development and progression of ARDS (Tschuschke et al., 2020; Ye et al., 2020). However, the transcriptomic studies targeting circRNAs in bronchoalveolar lavage fluid (BALF) exosomes of patients with ARDS caused by severe pneumonia have rarely been reported.

Exosomes are membrane vesicles that are released into the extracellular matrix after the fusion of polyvesicular bodies formed by cytoplasmic membrane invaginations and the cell membrane. Their diameter is 30–150 nm. They typically range in size from 30 to 150 nm and contain a distinct set of protein markers, including CD9, CD63, CD81, Alix, and TSG101, regardless of their cell source. However, their contents and quantity vary depending on the parental cells. As an important tool for information communication between cells, exosomes derived from different cells can precisely regulate the functions of target genes and proteins by carrying specific bioactive components (such as circRNA, LncRNA, miRNA, DNA, and membrane protein signaling molecules) (Tschuschke et al., 2020). In the mouse ALI model, exosomes secreted by alveolar macrophages were found to carry TNF and IL-1β into alveolar epithelial and endothelial cells, promoting the expression of cell adhesion molecule ICAM-1, the injury of endothelial cells, and the infiltration of polymorphonuclear leukocytes (PMN) in the lung and mediating the lung inflammatory injury of ALI (Ye et al., 2020). Exosomes loaded with miR-223 produced by alveolar macrophages can reduce lung inflammatory injury in ALI by inhibiting NF-κB (Zhang et al., 2019). These studies provide evidence for the potential application of exosome-loaded non-coding RNA to intervene in specific pathophysiological pathways of ALI. Despite this, there is a lack of research targeting circRNAs in BALF exosomes of patients with ARDS caused by severe pneumonia.

This study aimed to explore the expression of various circRNAs in BALF exosomes of patients with ARDS caused by severe pneumonia in the real world and identify potential regulating targets during the progression of ARDS. CircRNA microarrays were performed based on the discovery cohort of ARDS patients (n = 4) and healthy subjects (n = 4) to screen differentially expressed circRNAs in BALF exosomes. Afterward, several differentially expressed circRNAs were validated by real-time quantitative PCR (RT-qPCR), and ceRNA and function analyses were performed. Furthermore, circRNAs with the most significant difference were selected to evaluate the diagnostic value using receiver operating characteristic (ROC) curve analysis based on the validation cohort with a large sample size (n = 38, each group).

## Materials and methods

### Clinical sample selection

The case group consisted of hospitalized patients with ARDS caused by severe pneumonia who were admitted to the respiratory intensive care unit (RICU) of Shanghai East Hospital from May 2020 to May 2021. Diagnosis of ARDS was based on the Berlin definition 2012 (ARDS Definition Task Force et al., 2012). Meanwhile, healthy volunteers were recruited as the control group in this study, with an age of 18–75 years. All subjects who failed to tolerate bronchoscopy or were accompanied by contraindications to bronchoscopy were excluded. BALF and plasma samples of ARDS patients were simultaneously collected within 48 h after diagnosis. In the control group, BALF samples were also obtained from healthy volunteers. All samples were stored at −80°C.

### Study design

**Figure 1:**
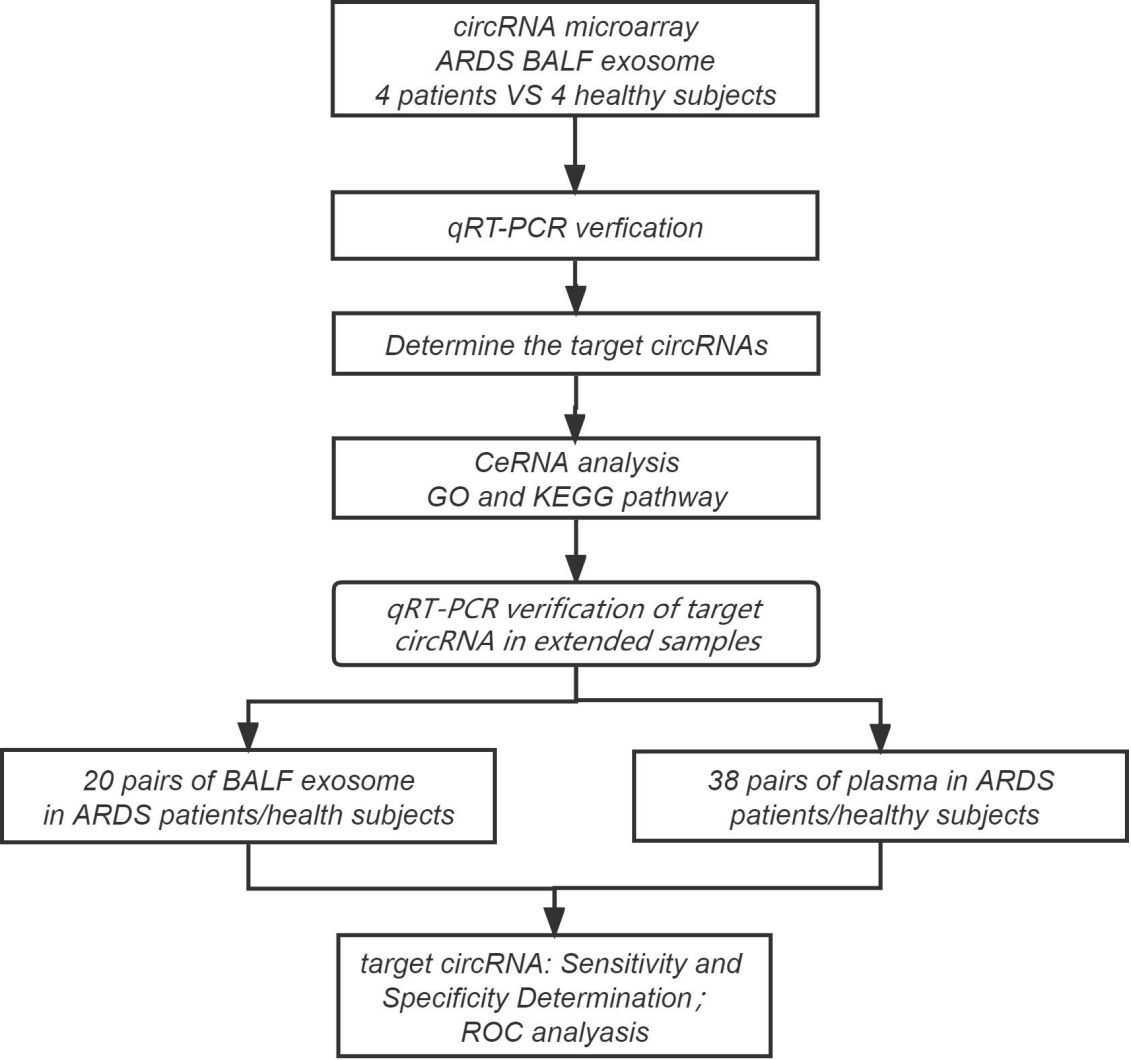
Experimental design and flowchart for this study.

Four ARDS patients and four healthy subjects were screened for the following circRNA microarray analysis as the discovery cohort. The clinical data of these four patients, including age, sex, C-reactive protein (CRP) level, interleukin-6 (IL-6) level, arterial partial pressure of oxygen/fraction of inspired oxygen (PaO_2_/FiO_2_) ratio, pathogen detection in BALF, and whether received invasive mechanical ventilation therapy, were obtained. First, circRNA microarray was performed to obtain the expression profile of circRNAs in BALF exosome of the case and control groups, and then the differentially expressed circRNAs between the two groups were selected with the threshold value of fold change ≥| ± 2|. Therewith, the top 9 differentially expressed circRNAs were validated using RT-qPCR, and three circRNAs with significant differences were selected for ceRNA, Gene Ontology (GO), and the Kyoto Encyclopedia of Genes and Genomes (KEGG) analyses. Furthermore, two circRNAs that were closely associated with ARDS progression were selected for the following study with a larger sample size, including 38 pairs of ARDS patients (case group) and healthy subjects (control group). The plasma of the 38 pairs of individuals and the BALF exosome of 20 of the 38 pairs of individuals were simultaneously collected to detect the two circRNAs using RT-qPCR. The clinical data of these subjects, including age, sex, respiratory rate (RR), CRP level, IL-6 level, procalcitonin (PCT) level, PaO_2_/FiO_2_ ratio, whether high flow and non-invasive ventilation or intubation and invasive ventilation, Acute Physiology and Chronic Health Evaluation System (APACHE II) score, Sequential Organ Failure Assessment (SOFA) score, and 28-day mortality, were summarized. ROC curve analysis was performed to evaluate the diagnostic value of target circRNAs in acute ARDS according to the sensitivity, specificity, and area under the curve (AUC). The concise research flowchart is displayed in **Figure 1**.

The study conformed to the 1964 Declaration of Helsinki and its subsequent amendments, and all subjects provided signed informed consent for the study. This study was approved by the Ethics Committee of the East Hospital, Tongji University (approval number 2022008).

### Bronchoscopy and BALF collection and detection

BALF was collected according to the Chinese Expert Consensus on Pathogen Detection in Bronchoalveolar Lavage for Pulmonary Infectious Diseases (2017 edition) and Guidelines for Clinical Application of Bronchoalveolar Lavage Cytology in Interstitial Lung Disease (ATS 2012 edition) (Meyer et al., 2012; Chinese Thoracic Society, 2017). Bronchoscopy was performed under vein inhalation combined anesthesia or topical anesthesia with 2% lidocaine in all patients after excluding contraindications. BALF measuring 20 mL was collected by negative pressure aspiration after fractional injection of the same amount of saline into the target segmental bronchi and stored at −80°C until use.

### Isolation and identification of BALF exosome

**Figure 2:**
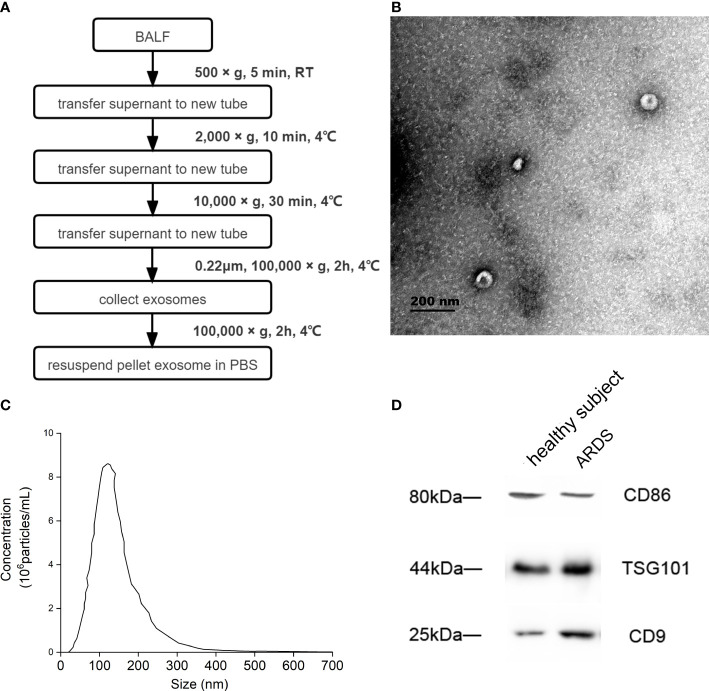
Identification of BALF exosome. **(A)** Isolation and identification of BALF exosomes from ARDS patients and healthy subjects. Isolation process of BALF exosomes. **(B)** The morphology of the isolated exosome detected by Hitachi 7650 transmission electron microscope from one ARDS patient. **(C)** The particle size and concentration of exosome measured using nanoparticle tracking analysis of the same ARDS patient. **(D)** The properties and possible cellular sources of exosome identified using Western blotting from the same ARDS patient. BALF, bronchoalveolar lavage fluid; ARDS, acute respiratory distress syndrome.

BALF exosome was isolated by ultracentrifugation. Briefly, 20 mL of BALF was centrifuged at 500 g for 5 min to remove cells. The supernatant was transferred to a new centrifuge tube and then centrifuged at 2,000 *g* for 10 min and successively 10,000 *g* for 30 min. Afterward, the large vesicles were filtered with a 0.22-μm filter membrane, and 1 mL of the supernatant was centrifuged at 100,000 *g* for 70 min to obtain the exosomes in the precipitation. Finally, the exosome was resuspended in 100 μL of phosphate-buffered saline (PBS). Exosomes from the same sample were evenly divided into three equal parts and stored at −80°C. The detailed flow is shown in **Figure 2A**. A transmission electron microscope (TEM; Hitachi 7650, Tokyo, Japan) was used to identify the isolated exosome, and nanoparticle tracking analysis (NTA; NanoSight NS300, Malvern Technologies, Malvern, UK) was applied to measure the particle size and concentration of exosome. The concentration of exosomes ranged from 10^6^ to 10^7^ particles per mL.

### Western blotting analysis

Protein was isolated by lysis of 100 μL of BALF exosome using radioimmunoprecipitation assay (RIPA) lysis buffer (CST, Danvers, MA, USA) and quantified by bicinchoninic acid (BCA) protein quantitation method (Thermo Fisher Scientific, Waltham, MA, USA). Then, the protein underwent resolution on sodium dodecyl sulfate–polyacrylamide gel electrophoresis (SDS-PAGE) gel and transferred to polyvinylidene difluoride (PVDF), followed by membrane blocking with 5% bovine serum albumin and reaction with anti-CD9, anti-TSG101, and anti-CD86 antibodies (CD9: 13403 (1:1,000), CST; TSG101: ab125011 (1:1,000), Abcam, Cambridge, UK; CD86: 91882 (1:1,000), CST) overnight at 4°C. Horseradish peroxidase-labeled anti-rabbit HRP (IgG HampL) or anti-mouse HRP (IgG HampL, Abcam) secondary antibodies were incubated for 1 h at 37°C. Protein expression was detected by enhanced chemiluminescence (ECL; Thermo) kit and analyzed using ImageJ software.

### Exosome RNA extraction and circRNA microarrays

Exosome RNA was extracted using TRIzol LS reagent (Thermo) according to the manufacturer’s instructions. The concentration and purity of RNA were detected by NanoDrop 1000, and the eligible RNA was determined with absorbance values at 1.8–2.0 for 260/280 and 1.8–2.0 for 260/230. The yield of the extracted RNA ranged from 844.65 to 2,389 ng. Linear RNA was removed with RNase to enrich circRNAs. The circRNAs underwent amplification and transcription into cy3-labeled cRNA (Arraystar Super RNA Labeling Kit, Arraystar Inc., Rockville, MD, USA). Hybridization was then performed with Arraystar Human circular RNA microarray (13,617, 8 × 15K), and data extraction was carried out using the Agilent Scanner G2505C (Jamul, CA, USA).

### RT-qPCR detection

Complementary DNA (cDNA) synthesis was performed by reverse transcription with 1 μg of total RNA using random primers (Superscript Reverse Transcription System, Invitrogen, Carlsbad, CA, USA). The following RT-qPCR was finished using SYBR Green assay (Arraystar, Rockville, MD, USA) on ABI 7900 system (Applied Biosystems, Foster City, CA, USA). β-Actin was used as the internal standard for circRNA expression. Fold changes in circRNA expression were calculated using the formula 2^−ΔΔCT^. The primer sequence is presented in **Table 1**.

**Table 1:** Primer sequences for circRNA RT-qPCR assay.

circBase ID	Primer sequence	Tm (°C)	Product size (bp)
β-Actin (human)	F:5′ GTGGCCGAGGACTTTGATTG3′ R:5′ CCTGTAACAACGCATCTCATATT3′	60	73
hsa_circRNA_066608	F:5′ TGCCCTATGGGATGAGAAC 3′ R:5′ ATGGCTGGCTCACTTGTCA 3′	60	101
hsa_circRNA_009128	F:5′ CTTCACCTCAGCATTACATTCA 3′ R:5′ GTCAAGGACTGGAGACCTCAA 3′	60	105
hsa_circRNA_104034	F:5′ AAAGAAGTCATGGATTGGGAA 3′ R:5′ CTTGTCAGGATCAATCACTAACA3′	60	114
hsa_circRNA_102759	F:5′ CCAGAATTAGAAAAAGAAAGCC 3′ R:5′ AACTTGCTGATTTCCCTGTAGA 3′	60	86
hsa_circRNA_104534	F:5′ GACGGTGGTCACGCCTGTT 3′ R:5′ CCTCTGCTGAAAGCTTGGGA3′	60	97
hsa_circRNA_002809	F:5′ CATTCCAGTTTTCCTGATGGT 3′ R:5′ ATTACTCTTCTTATTTGTGGCTTC3′	60	94
hsa_circRNA_102394	F:5′ CTGCACGTCTTCACCTTCG 3′ R:5′ CTGCTGCTTCCCGTTCTTAC 3′	60	161
hsa_circRNA_008201	F:5′ CAGACCTACCTTCAGTCAACAA3′ R:5′ GTGTGTGTAGCCATATTTTTCAT 3′	60	111
hsa_circRNA_042882	F:5′ AATACGAATGGCACCGCTTC 3′ R:5′ AGTAGTGAGGCCGCTTATAACC 3′	60	136
hsa_circRNA_008550	F:5′ TTTCAATGATGATGCTATGCTG 3′ R:5′ TTATGGATTTGGATGTGCTCG 3′	60	65

### Construction of ceRNA network and functional enrichment analysis

Two databases, TargetScan (http://www.targetscan.org/vert_72/) and miRanda (http://www.microrna.org/microrna/) were used to predict the associations of circRNA with miRNAs as well as the miRNA-targeted mRNAs. Afterward, the ceRNA network was constructed using cytoscape software (cytoscape 3.8.1 https://cytoscape.org/index.html). Subsequently, GO and KEGG pathway analyses based on mRNAs in the ceRNA network were, respectively, carried out using the topGO software package and cluster Profiler software package for R language.

### Statistical analysis

GraphPad Prism 8 and SPSS 25.00 software were respectively used for graphical and statistical analysis. Measurement data conforming to normal distribution were expressed as mean ± standard deviation (SD), and comparison between two groups was performed by Student’s *t*-test. Enumeration data were expressed as the number of cases and rate, and the qualitative data were expressed as frequency (%), which were all compared by χ^2^ test and Fisher’s exact test. The scatter plots and ROC curves were generated by GraphPad Prism 8. MedCalc 19.1 statistical software was used to analyze ROC curves and compare the AUCs. *p* < 0.05 was considered statistically different.

## Results

### Clinical characterization of the study subjects

Four ARDS patients in the discovery cohort were aged 53–69 years, including two carbapenemase-resistant Enterobacteriaceae pneumonia, one *Pneumocystis jirovecii* pneumonia, and one human cytomegalovirus pneumonia (**Table 2**). The demographic and clinical characteristics of ARDS patients (case group) and healthy subjects (control group) in the validation cohort with an expanded sample size (38 pairs) are shown in **Table 3**. ARDS of patients in the case group was caused by severe pulmonary infection, of which the etiological diagnosis was obtained based on bronchoscopy. The responsible pathogens of pulmonary infection in these patients included carbapenemase-resistant *Klebsiella pneumoniae* in 10 cases (26.31%), SARS-CoV-2 + *Aspergillus fumigatus* in nine cases (23.68%), *P. jirovecii* in seven cases (18.42%), *P. jirovecii +* human cytomegalovirus in five cases (13.15%), adenovirus in three cases (7.89%), *Legionella pneumophila* in two cases, and *Chlamydia psittaci* in two cases (5.26%) (**Table 4**). There were no significant differences in age and sex between the case and control groups, while the clinical characteristics, including RR, CRP, IL-6, PCT, PaO_2_/FiO_2_ ratio, whether high flow and non-invasive ventilation or intubation and invasive ventilation, APACHE II score, SOFA score, and 28-day mortality, revealed significant differences (*p* < 0.01, **Table 3**).

**Table 2:** The demographic and clinical characteristics of patients with acute respiratory distress syndrome (ARDS).

Characteristics	Case 1	Case 2	Case 3	Case 4
Age (years)	67	54	53	69
Sex	Male	Male	Female	Male
CRP (mg/L)	257.6	376.2	190.5	234.8
IL-6 (pg/mL)	187.8	1,321.6	576.9	678.3
PaO_2_/FiO_2_	140.3	136.2	167.3	158.4
Pathogen detection in BALF	CRKP	PJ	CMV	CRE
Invasive mechanical ventilation therapy	Yes	Yes	Yes	Yes

CRP, C-reactive protein; IL-6, interleukin-6; PaO_2_/FiO_2_, arterial partial pressure of oxygen/fraction of inspired oxygen; CRKP, carbapenemase-resistant Klebsiella pneumoniae; CRE, carbapenemase-resistant Escherichia coli; PJ, Pneumocystis jirovecii, CMV, human cytomegalovirus.

**Table 3:** The demographic and clinical characteristics of patients with acute respiratory distress syndrome (ARDS) and healthy subjects.

Characteristics	Case group (ARDS patients) (n = 38)	Control group (healthy subjects) (n = 38)	*p*-Value
Age (years)	54.26 ± 18.02	50.34 ± 19.27	>0.05
Male (%)	29 (82.85)	27 (77.14)	>0.05
RR (x/min)	24.16 ± 6.24	16 ± 3.61	<0.01
CRP (mg/L)	245.13 ± 38.53	<5	<0.01
IL-6 (pg/mL)	586.23 ± 178.35	10.38 ± 4.61	<0.01
PCT (ng/ml)	5.86 ± 4.07	<0.046 ± 0.015	<0.01
200 < P/F ≤ 300 (%)	7 (20.00)	0	<0.01
100 < P/F ≤ 200 (%)	10 (28.57)	0	<0.01
P/F ≤ 100 (%)	18 (51.42)	0	<0.01
High flow and non-invasive ventilation (%)	8 (22.85)	0	<0.01
Intubation and invasive ventilation (%)	27 (77.14)	0	<0.01
APACHE II score	17.31 ± 6.27	0	<0.01
SOFA score	7.81 ± 5.02	0	<0.01
28-day mortality	11 (31.43)	0	<0.01

RR, respiratory rate; CRP, C-reactive protein; IL-6, interleukin-6; PCT, procalcitonin; P/F, arterial partial pressure of oxygen/fraction of inspired oxygen; APACHE II score, Acute Physiology and Chronic Health Evaluation System; SOFA score, Sequential Organ Failure Assessment.

**Table 4:** Constituent ratio of responsible pathogens in ARDS patients of case group.

Case group (n = 38)	No.	Ratio. (%)
*Pneumocystis jirovecii*	7	18.42%
*P. jirovecii* + human cytomegalovirus	5	13.15%
*Legionella pneumophila*	2	5.26%
SARS-CoV-2 + *Aspergillus fumigatus*	9	23.68%
Adenovirus	3	7.89%
*Chlamydia psittaci*	2	5.26%
Carbapenemase-resistant *Klebsiella pneumoniae*	10	26.31%

### Identification of BALF exosome

The BALF of a patient with ARDS caused by severe pneumonia was selected to complete the identification of exosomes. The morphology of extracellular vesicles was observed using TEM, and the exosome presented a tea tray-like structure, which was consistent with the morphological characteristics of the exosome (**Figure 2B**). Then, NTA showed that the particle size distribution of the isolated BALF exosome ranged from 50 to 150 nm, and the concentration of exosomes ranged from 10^6^ to 10^7^ particles per mL (**Figure 2C**). Furthermore, the properties of these extracellular vesicles were identified by the expression of CD9 and TSG101 using Western blotting, and the results proved that these extracellular vesicles were exosomes. Subsequently, the possible cellular origin of these exosomes was examined. To identify whether the source of BALF exosomes was associated with alveolar macrophages, CD86 was tagged with anti-CD86 antibody, and alveolar macrophages were found to be possibly the main source of these exosomes (**Figure 2D**).

### Screening and validation of differentially expressed circRNAs in BALF exosome

**Figure 3:**
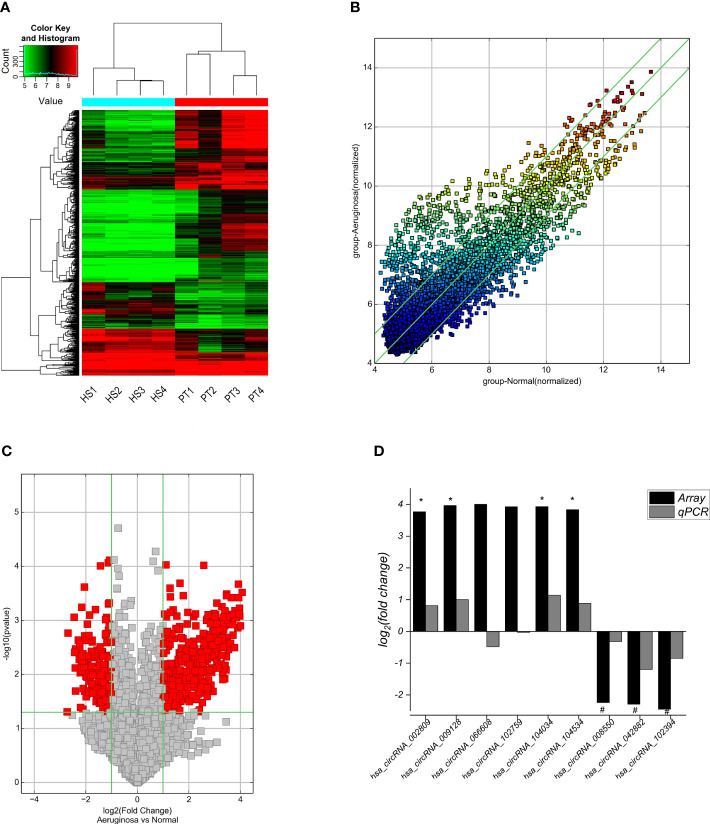
Cluster analysis and RT-qPCR validation of differentially expressed circRNAs in the BALF exosomes from ARDS patients and healthy subjects. **(A)** The cluster analysis plot of differentially expressed circRNAs; red represents upregulated circRNAs, and green represents downregulated circRNAs. **(B)** The scatter plot shows differences in circRNA expression in BALF exosomes from case–control groups. The x- and y-axes in the figure represent the mean normalized signal values (log2 scaled) of the sample group. The green fold line represents± 2|-fold, and therefore, the icon above or below the green line represents more than -fold upregulation or downregulation. **(C)** Volcano plot visualization shows differentially expressed circRNAs. Vertical bars show fold change >| ± 2| (log2 fold change); horizontal green lines represent *p* = 0.05, −log10 (*p*-value), and red dots in the figure represent circRNAs with significant expression differences in statistics. **(D)** RT-qPCR validation of the nine circRNAs with the most significant differences reveals that seven circRNAs with significant differences between groups are consistent with the gene microarray screening results, with four circRNAs significantly upregulated and three significantly downregulated in the case group. BALF, bronchoalveolar lavage fluid; ARDS, acute respiratory distress syndrome.

A total of 13,228 circRNAs were detected by circRNA microarray, among which 629 circRNAs were differentially expressed in BALF exosomes between ARDS patients and healthy subjects (fold change **>**| ± 2|, *p* < 0.05), including 430 upregulated and 199 downregulated circRNAs in the BALF exosomes from ARDS patients compared with healthy subjects. The clustering heatmap (**Figure 3A**), scatter plot (**Figure 3B**), and volcano plot (**Figure 3C**) all visually displayed these differentially expressed circRNAs. Subsequently, according to the following criteria for selecting differentially expressed circular RNAs from a circular RNA microarray (fold change >| ± 4|, *p*-value <0.05, and raw signal intensity greater than 1,000), the top 9 differentially expressed circular RNAs were selected for subsequent validation by RT-qPCR. The results confirmed four upregulated circRNAs (hsa_circRNA_009128, hsa_circRNA_104034, hsa_circRNA_104534, and hsa_circRNA_002809) and threedownregulated circRNAs (hsa_circRNA_102394, hsa_circRNA_042882, and hsa_circRNA_008550) in BALF exosomes of the case group compared with the control group, consistent with the microarray results (**Figure 3D**).

### Construction of ceRNA network and functional enrichment analysis of target genes

**Figure 4:**
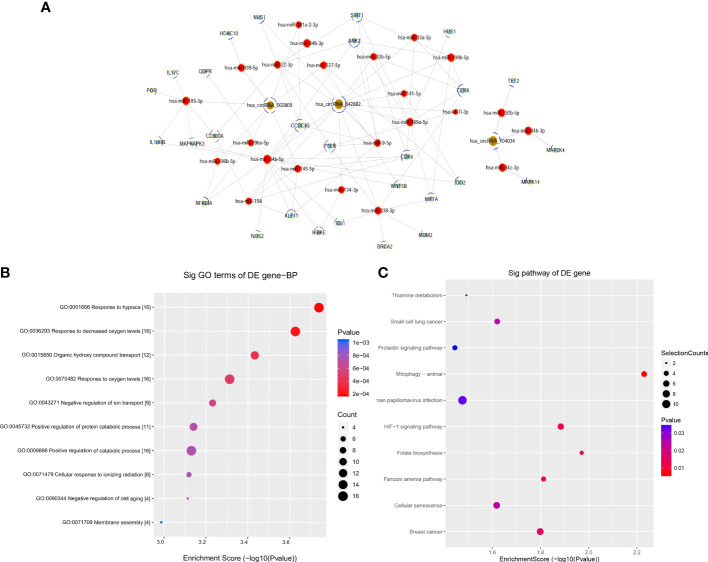
Construction of ceRNA network and functional enrichment analysis of target genes. **(A)** CircRNA–miRNA–mRNA network prediction and analysis based on the main target genes of hsa_circRNA_002809, hsa_circRNA_042882, and hsa_circRNA_104034, with yellow representing circRNA, red representing microRNA, and blue representing mRNA. **(B, C)** GO **(B)** and KEGG **(C)** pathway analyses were performed on the predicted target genes based on ceRNA analysis, and the results show the biological processes and signaling pathways of top 10 significant difference, with the horizontal axis representing −log10(*p*) and the vertical axis representing GO items or KEGG pathways. GO, Gene Ontology; KEGG, Kyoto Encyclopedia of Genes and Genomes; ceRNA, competitive endogenous RNA.

Based on the above validation, three target genes with the largest fold change in expression and the smallest *p*-value, including hsa_circRNA_002809, hsa_circRNA_042882, and hsa_circRNA_104034, further underwent ceRNA analysis. The network of circRNA–miRNA–mRNA contained 180 targeted miRNAs and 356 targeted mRNAs, along with key circRNA–miRNA–mRNA regulatory axes such as hsa_circRNA_0042882-hsa-miR-199a-5p/hsa-miR-199b-5p-HIF1A, hsa_circRNA_0042882-5p-hsa-miR-34b-5p-NFKBIA, hsa_circRNA_104034-hsa-miR-34b-3p-MAP2K4, and hsa_circRNA_002809-hsa-miR-939-5p-HDAC10 (**Figure 4A**). Furthermore, GO analysis showed that these targeted mRNAs were mainly enriched in GO terms of response to hypoxia and response to decreased oxygen signaling levels, and the top 10 GO terms in biological processes are indicated in **Figure 4B**. In addition, KEGG signaling pathway analysis displayed the top 10 signaling pathways related to the above target genes, and these target genes were closely associated with the HIF-1 signaling pathway and small cell lung cancer (**Figure 4C**).

### Prediction of diagnostic value of circRNAs in ARDS

**Figure 5:**
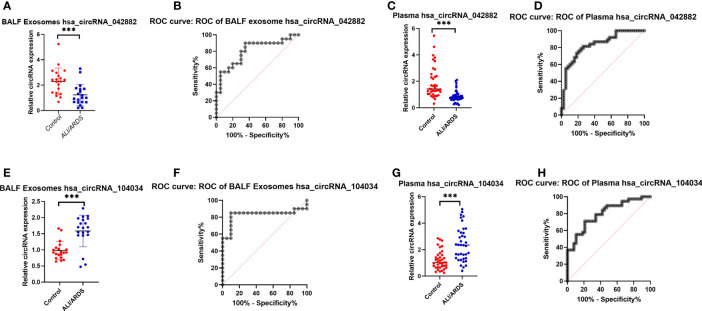
Expression levels and ROC curve analysis of hsa_circRNA_042882 and hsa_circRNA_104034 in BALF exosomes and plasma of ARDS patients and healthy subjects. **(A, C)** Expression levels of hsa_circRNA_042882 and hsa_circRNA_104034 in BALF exosomes of ARDS patients and healthy subjects. **(B, D)** ROC curve analysis of hsa_circRNA_042882 and hsa_circRNA_104034 in BALF exosomes of ARDS patients and healthy subjects. **(E, G)** Expression levels of hsa_circRNA_042882 and hsa_circRNA_104034 in plasma of ARDS patients and healthy subjects. **(F, H)** ROC curve analysis of hsa_circRNA_042882 and hsa_circRNA_104034 in plasma of ARDS patients and healthy subjects. The *** in the figure represents *p* < 0.001, indicating significant difference in the expression of these circRNAs in BALF exosomes and plasma between normal subjects and ARDS patients. ROC, receiver operating characteristic; BALF, bronchoalveolar lavage fluid; ARDS, acute respiratory distress syndrome.

According to the results of the ceRNA analysis, two circRNAs (circ042882 and circ104034) with the most significant expression differences in BALF exosomes between the case group and the control group were selected for further study with a larger sample size. The results showed that the expression of circ042882 in BALF exosomes of ARDS patients was significantly lower than that of the control group, with an AUC of 0.8050, sensitivity of 90%, and specificity of 65% in the diagnosis of ARDS (*p* < 0.001, **Figures 5A, B**); the expression of circ104034 was significantly higher, with an AUC of 1, sensitivity of 85%, and specificity of 90% in the diagnosis of ARDS (*p* < 0.001, **Figures 5C, D**). Similarly, the expression of circ042882 in the plasma of ARDS patients was prominently lower than that in the control group, with an AUC of 0.835, sensitivity of 76.32%, and specificity of 78.95% in the diagnosis of ARDS (*p* < 0.001, **Figures 5E, F**); the expression of circ104034 was distinctly higher, with an AUC of 0.799, sensitivity of 71.05%, and specificity of 78.95% in the diagnosis of ARDS (*p* < 0.001, **Figures 5G, H**). In the validation experiment with an expanded sample size, three circular RNAs (circ104534, circ002809, and circ009128) that showed significant differential expression in BALF exosomes of ARDS patients were tested, of which the results showed no statistically significant difference (*p* > 0.05) in the expression of these three circular RNAs in BALF exosomes between ARDS patients and healthy controls (**Supplementary Figure 1**). These results suggested that circ042882 and circ104034 in both BALF and peripheral plasma were promising potential diagnostic markers in the acute phase of ARDS.

## Discussion

With the advanced understanding of the clinical and pathological features of ARDS, the hallmark pathological changes of ARDS, from diffuse alveolar injury in the acute phase to granulomatous tissue hyperplasia and lung tissue fibrosis in the later stages, have also been gradually recognized (Ashbaugh et al., 1967). However, significant heterogeneity is reported in ARDS patients who meet its clinical consensus criteria (Estenssoro et al., 2002), and biomarker measurement is more objective relative to the subjective effects existing in clinical diagnosis (Viswan et al., 2017). Regrettably, effective laboratory indicators are lacking for the diagnosis and prognosis of ARDS; thus, searching for biomarkers with high sensitivity and specificity is one of the goals of ARDS research. Herein, we concentrated on circRNAs in the BALF exosome and plasma from patients with ARDS caused by severe pneumonia and aimed to discover reliable biomarkers for the diagnosis of acute ARDS.

Based on high-throughput sequencing technology, Memczak S et al. (Memczak et al., 2013) discovered the stable presence of a large number of circRNAs in eukaryotic cells, which initiates a new chapter for the exploration of the roles of circRNAs in different diseases. CircRNAs are endowed with high conservatism and stability due to the unique closed loop structure avoiding cleavage by RNA exonuclease (Xiao et al., 2020); thus, circRNAs are considered as priority alternative genes for biomarker research. Similar to previous animal experiments, we also found that hundreds of circRNAs were differentially expressed in the BALF exosomes from ARDS patients compared with healthy subjects using circRNA microarray technology, suggesting that circRNAs exerted pivotal roles in the progression of ARDS.

According to further verification by RT-qPCR, the three circRNAs with the most significant expression differences (hsa_circRNA_002809, hsa_circRNA_042882, and hsa_circRNA_104034) were selected for ceRNA analysis and functional enrichment analysis of target genes. Based on the network of circRNA–miRNA–mRNA, we focused on two important regulatory axes of hsa_circ_0042882: hsa_circ_0042882-hsa-mir-199a-5p/hsa-mir-199b-5p-HIF1A and hsa_circ_0042882-5p-hsa-mir-34b-5p/hsa-mir-34b-5p-NFKBIA. It is well-known that the core transcription factor HIF-1 is widely expressed in hypoxic tissues and involved in inflammation, cell apoptosis, and pulmonary fibrosis by regulating the expression of a series of hypoxic-adaptive-related genes (Jin et al., 2020). Notably, a significant positive feedback pro-inflammatory effect has been proven during the progression of ARDS (Lee et al., 2004; Li et al., 2018). Plenty of inflammatory factors are capable of inducing the overexpression of HIF-1; in turn, upregulated HIF-1 further stimulates the release of inflammatory factors, thereby enhancing the inflammatory response and aggravating lung injury (Chow et al., 2003; Suresh et al., 2019). In addition, NFκBIA acts as the upstream of the NF-κB signal pathway. Previous studies have demonstrated that the activation of the nuclear regulatory factor NF-κB can be induced by superfluous reactive oxygen species (ROS), and excessive production of ROS has been indicated to be closely implicated in the occurrence and progression of ALI (Bonello et al., 2007; Zhang et al., 2011). Moreover, studies have shown that a hypoxic environment promotes the production of ROS in mitochondria, and then increased ROS can upregulate HIF-1 expression, which leads to sustained high levels of oxidative stress. Interestingly, HIF-1 also possesses the ability to inhibit oxidative phosphorylation and ROS production, suggesting that the ROS-mediated negative feedback loop of HIF-1 regulation may also participate in the progression of ALI (Papandreou et al., 2006; Prabhakar et al., 2007). Consistently, our results of KEGG analysis also showed that the circRNA-related target genes, such as *HIF1A* and *NFKBIA*, were mainly associated with two signaling pathways: HIF-1 signaling pathway and small cell lung cancer. Furthermore, for hsa_circRNA_104034 and hsa_circRNA_002809, we concentrated on the regulatory axis of hsa_circRNA_104034-hsa-miR-34b-3p-MAP2K4 and hsa_circRNA_002809-hsa-miR-939-5p-HDAC10, respectively. It has been found that the MAPK signaling pathway is significantly activated in the acute phase of ARDS. The p38 MAPK pathway is involved in LPS-induced IL-6 secretion, and simultaneous stimulation of the p38 MAPK and NF-κB signaling pathways can also induce the expression and release of IL-6 (Craig et al., 2000; Schnyder-Candrian et al., 2005). Additionally, histone deacetylases (HDACs) are reported to be involved in various diseases including cancer and inflammatory diseases by regulating the expression of various pro- and anti-inflammatory genes *via* NF-κB signaling pathways (Ashburner et al., 2001; Ziesché et al., 2013; Leus et al., 2016). All these findings indicated that these differentially expressed circRNAs might be involved in the progression of ALI/ARDS through regulating miRNAs and target genes.

In order to further verify the reliability of the results of the preliminary microarray detection, we further expanded the sample size in the subsequent experiments. After the differential expression of several different circRNAs was verified, the results also proved that in the validation cohort, hsa_circRNA_042882 was lowly expressed and hsa_circRNA_104034 was highly expressed in ARDS patients compared with healthy subjects, which were consistent with the results of discovery cohort. Importantly, we found that hsa_circRNA_042882 and hsa_circRNA_104034 in both BALF exosomes and peripheral plasma were promising candidates as diagnostic biomarkers for ARDS, as indicated by the ROC curve analysis and sensitivity/specificity results. These data confirmed the potential diagnostic value of hsa_circRNA_042882 and hsa_circRNA_104034 for ARDS at the acute phase.

This study has some limitations. First, this study only included patients with ARDS caused by severe pneumonia, while patients with ARDS caused by other etiologies, such as extra-pulmonary sepsis, acute trauma, or poisoning, were not recruited. Therefore, the above results could only reflect the expression characteristics of circRNAs in patients with ARDS caused by severe pneumonia. Due to the heterogeneity of ARDS disease, it is necessary to verify the expression of the above circRNAs in ARDS cases caused by other etiologies in future studies. Second, due to the limited sample size in this study, further studies with a large sample size should be performed to verify the stability and reliability of these results. In addition, due to the lack of stratified analysis according to the infected pathogenic microorganisms, this study cannot distinguish the differences in circRNA expression in patients with ARDS caused by different pathogen infections. Thus, the expression of circRNAs should be detected and analyzed in BALF and peripheral blood from different patients with ARDS caused by bacteria, fungi, viruses, and atypical pathogenic microorganisms. During the implementation of this study, TSG101 and CD9 were selected as positive markers to verify the presence of exosomes by Western blotting. However, a negative marker, such as calnexin, was not included. According to the relevant literature, β-actin was selected as an internal reference for RT-qPCR of circular RNA in this study. In future research, RNAhsa_circ_0000284 and hsa_circ_0000471 may be better options, as they are reported to be more stable. These improvements in study design will be beneficial for discovering more reliable and generally applicable biomarkers, as well as obtaining reliable diagnostic biomarkers in ARDS patients with different etiologies.

## Conclusions

In conclusion, this study discovered two circRNAs, hsa_circRNA_042882 and hsa_circRNA_104034, that were validated to be closely associated with the progression of ARDS, and both were regarded as promising diagnostic biomarkers for patients with ARDS caused by severe pneumonia. It is necessary to investigate the function and mechanism of these two circRNAs in the following study, which may contribute to the search for effective targets for intervening in the progression of ARDS and improving its prognosis.

## Data availability statement

The datasets presented in this study can be found in online repositories. The names of the repository/repositories and accession number(s) can be found below: https://www.ncbi.nlm.nih.gov/, GSE217763.

## Ethics statement

The studies involving human participants were reviewed and approved by the Ethics Committee of the East Hospital. The patients/participants provided their written informed consent to participate in this study.

## Author contributions

HS, FW, and QL designed the paper. HS and WG drafted the manuscript. HS, WG, RC, SC, and XG are involved in the clinical care and management of the patients. HS and WG contributed equally to this work. All authors agree to be accountable for all aspects of the work. All authors contributed to the article and approved the submitted version.

## Supplementary material

Supplementary Figure 1 Expression levels and ROC curve analysis of hsa_circRNA_104534, hsa_circRNA_002809 and hsa_circRNA_009128 in BALF exosomes between ARDS patients and healthy subjects. **(A, C, E)**: Expression levels of hsa_circRNA_104534, hsa_circRNA_002809, and hsa_circRNA_009128 in BALF exosomes between ARDS patients and healthy subjects; **(B, D, F)**: ROC curve analysis of hsa_circRNA_104534, hsa_circRNA_002809 and hsa_circRNA_009128 in BALF exosomes between ARDS patients and healthy subjects.
