# Supplementary figures and images for: CircRNAs in BALF exosomes and plasma as diagnostic biomarkers in patients with acute respiratory distress syndrome caused by severe pneumonia
Source: Front Cell Infect Microbiol. 2023 Aug 22;13:1194495. doi: 10.3389/fcimb.2023.1194495 (PMC10477665; doi:10.3389/fcimb.2023.1194495)

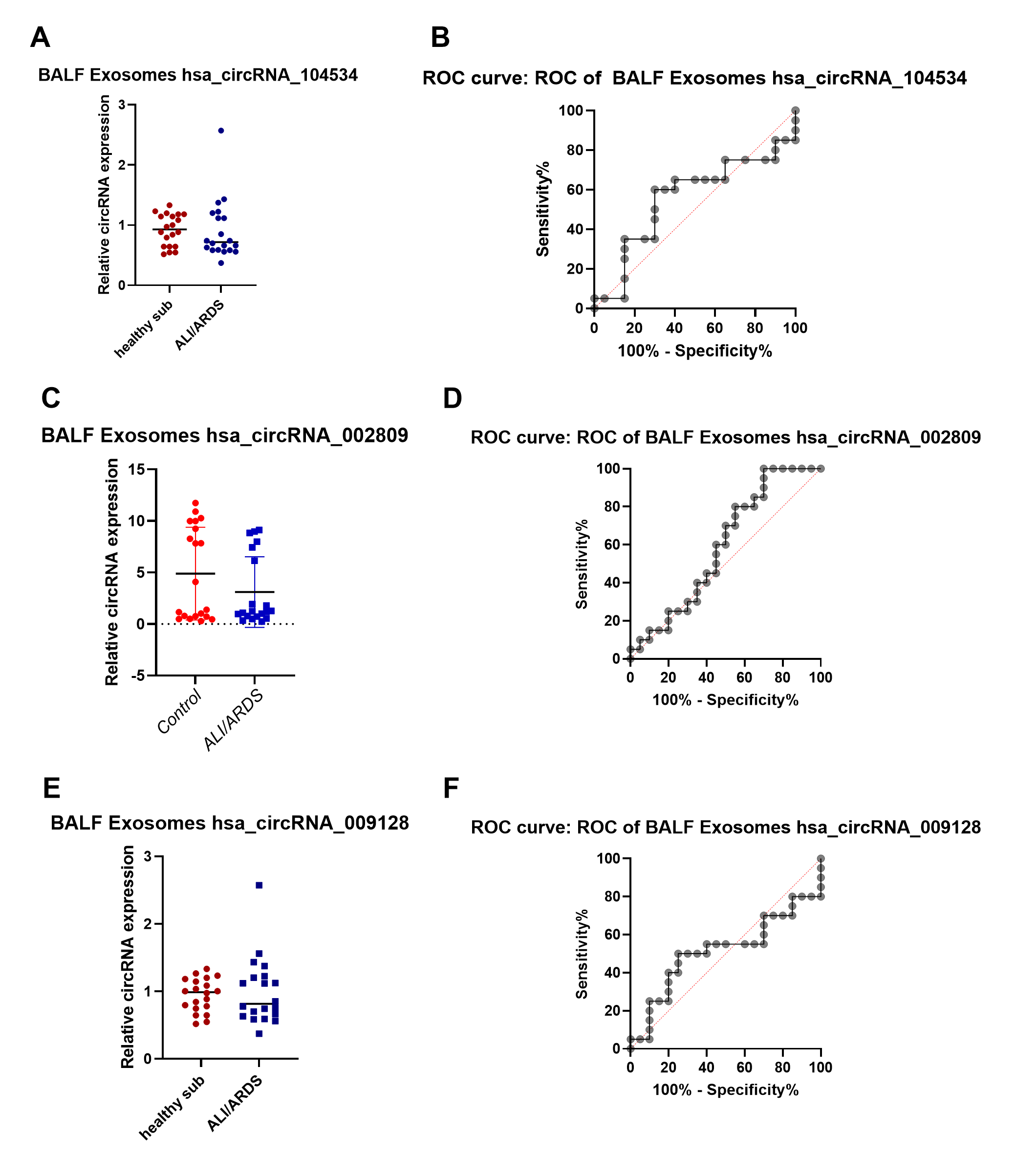

Supplement: Supplementary Figure 1 — Expression levels and ROC curve analysis of hsa_circRNA_104534, hsa_circRNA_002809 and hsa_circRNA_009128 in BALF exosomes between ARDS patients and healthy subjects. (A, C, E): Expression levels of hsa_circRNA_104534, hsa_circRNA_002809, and hsa_circRNA_009128 in BALF exosomes between ARDS patients and healthy subjects; (B, D, F): ROC curve analysis of hsa_circRNA_104534, hsa_circRNA_002809 and hsa_circRNA_009128 in BALF exosomes between ARDS patients and healthy subjects. [file Image_1.tif]
